# Prevalence and associated factors of medication-related problems among epileptic patients at ambulatory clinic of Mettu Karl Comprehensive Specialized Hospital: a cross-sectional study

**DOI:** 10.1186/s40545-022-00468-2

**Published:** 2022-10-27

**Authors:** Firomsa Bekele, Tadele Mamo, Ginenus Fekadu

**Affiliations:** 1 Department of Pharmacy, College of Health Science, Mattu University, Mattu, Ethiopia; 2 Department of Mechanical Engineering, College of Engineering and Technology, Mattu University, Mattu, Ethiopia; 3 Department of Pharmacy, College of Health Science, Wollega University, Nekemte, Ethiopia; 4 School of Pharmacy, Faculty of Medicine, The Chinese University of Hong Kong, Shatin, N.T, Hong Kong, China

**Keywords:** Drug-related problem, Epilepsy, Associated factors, Ethiopia

## Abstract

**Background:**

Despite appropriate treatment of epilepsy, the treatment outcome is poor in developing country. Therefore, the study was aimed to identify the magnitude and associated factors of epileptic patients at ambulatory clinic of south western Ethiopian hospital.

**Methods:**

A hospital-based cross-sectional study was conducted on epileptic patients who had follow-up at Mettu Karl Comprehensive Specialized Hospital (MKCSH). Data collection was done through patient interview and medical charts review. The pharmaceutical care network Europe foundation classification system was used to assess the drug therapy problem and Naranjo algorithm of adverse drug reaction was employed to identify the probability of adverse drug reaction. The data were analyzed by SPSS version 23 after data were entered by *Epidemiological Information ****(***Epi Info) 7.2.1. The multivariable logistic regressions were utilized and *P* < 0.05 was used to declare association.

**Results:**

Over the study period, more than half of the participants 172(57.7%) were males. The magnitude of drug-related problems was found to be 164(55.0%). Among epileptic patients about 323 drug-related problems (DRPs) were identified on average, 1.084 drug-related problems (DRPs) per patient. The widely occurred types of drug-related problems were needs additional drug therapy 72(22.29%), dose too low 52(16.09%) and dose to high which accounts 50(15.48%). Regarding the predictors of drug-related problems, being a female (AOR = 3.57, 95% CI:1.85–6.88, *P* ≤ 0.001), having frequent seizures (AOR = 2.47, 95% CI%:1.33–4.61, *P* = 0.004) and the presence of poly-pharmacy (AOR = 3.57, 95% CI: 1.49–8.5, *P* = 0.004) were predictors of drug-related problems.

**Conclusion:**

More than half of the patients had a drug therapy problem. Number of medications taken by the patients, gender and the seizure frequency had a significant association with occurrence of drug therapy problems (DTPs). Therefore, the pharmaceutical care in general and drug information services in particular should be established to hinder any undesirable medication effects in our study area.

## Background

Epilepsy is one of the neurologic disorders that is manifested as a frequent attacks of the seizures affecting about 50 million people globally in which more than three-fourths of them residing in developing countries [1–3]. In Ethiopia the prevalence of epilepsy is 64 per 100,000 populations [2].

The goal of epilepsy treatment is to control seizures without drug-related problems (DRPs) [4]. The epilepsy drug therapy should be individualized to reduce any drug-related problems [1]. However, almost all of epileptic patients have no access to the appropriate treatment in low-resource countries [2]. In south western Ethiopia, about two-thirds of the epileptic patients had had uncontrolled seizures due to inappropriate therapy [5]. Therefore, the drug-related problems should be reduced to enhance the treatment outcome of epileptic patients [6].

Despite appropriate treatment of epilepsy, the treatment outcome is poor in developing country [7]. This could be due to the complexity of the pharmacokinetics profiles of AEDs and the probability of the drugs to inhibit/induce the most metabolizing enzymes that pose difficulty in delivering appropriate dose of the drugs [1].

Non-adherences to anti-epileptic drugs (AEDs) were the common drug therapy problems that hinder the treatment outcomes of the epileptic patients. Hence, the hindrances to patient’s adherence to their treatment should studied at large to reduce poor treatment outcomes [3].

Different factors were associated with the occurrence of drug-related problems (DRPs) in epileptic patients that includes presence of poly-pharmacy, the presence of comorbidity, female sex and history of hospital admission [8, 9].

The occurrences of medication-related problems might lead to unsuccessful control of the seizure, hospitalization, increased health care cost, decreased quality of life [3, 9, 10]. Besides, the mortality rate is increased secondary to medication therapy problems [10, 11].

In Ethiopia, about 70% of the epileptic patients had encountered at least one drug-related problems [12]. The previous study only reports the magnitudes of the drug-related problems and they primarily focused on the assessment of adherence, and there is a scanty of studies on the associated factors of medication-related problems. Therefore, the study aimed to identify the predictors of medication-related problems among epileptic patients at ambulatory care.

## Patients and methods

### Study area, design and period

A cross-sectional study was conducted at Mettu Karl Comprehensive Specialized Hospital (MKCSH) from February 12, 2020 to August 11, 2020. MKCSH is found in Mattu town, South West Oromia, Ethiopia which is found at 600 km from Finfinne. There are different wards and clinics within MKCSH; those include internal medicine ward, surgery ward, pediatric ward, gynecology and obstetrics ward, antenatal clinic, dental clinics, tuberculosis clinic, anti-retro viral therapy clinic and ophthalmic clinic.

### Inclusion criteria

Adult patients (age ≥ 18 years) with the diagnosis of the epilepsy who have been on regular follow- up for at least 2 years with complete registration charts who were on drug therapy or who need drug therapy during study period and with more than 48 h of length of stay were included.

### Exclusion criteria

The epileptic patients who had not prescribed with anti-epileptic drugs, follow-up period of less than 2 years, seriously ill to complete the interview, refused to give consent, and those with incomplete medical records were excluded.

### Study variables and outcome endpoints

Medication-related problem was the primary outcome. Adverse drug reaction (ADR) was assessed by using the Naranjo algorithm of the ADR probability scale [13]. Hill–Bone Compliance to High Blood Pressure Therapy Scale was used to measure medication adherence [14]. For this study purpose, we had used a 9-item medication-taking sub-scale. Each item is a four-point Likert-type scale (none of the time, some of the time, most of the time, and all the time). The median split was used and dichotomies into adherent and non-adherent to the treatment. The pharmaceutical care network Europe guideline was used to assess the presence of medication-related problems. The work has been reported in line with the strengthening the reporting of cohort studies in surgery (STROCSS) criteria [15].

### Sample size and sampling technique

Single population proportion formula was used to calculate the required sample size by considering the magnitude of the problem *P* = 0.608 [5].

The sample size (*n*) was calculated as:

$$n \, = \frac {z^{{2}} p({1} - p)} {{{d}}^{{2}}},$$

where *n* = the desired sample size, *Z* = level of significance at 95% confidence interval which is 1.96, *P* = magnitude of problem, *q* = 1-*p, d* = margin of error which is 0.05, $n = \frac{ \left( {{1}.{96}} \right)^{{2}} \left( {0.{6}0{8}} \right)(0.{392})} {\left( {0.0{5}} \right)^{{2}}} = {366},$ ${\text{nf }} = \frac{n}{1 + n/N} = \frac{n \times N}{n+N},$ where nf = adjusted sample size, *n* = sample from infinite population, *N* = population size,

$$\mathrm{nf}=\frac{366\times 1040}{366+1040 }=271.$$

By adding 10% contingency, the final sample size becomes 298. A simple random sampling was used to include study participants.

### Data collection process and management

A semi-structured data collection tool was prepared to collect the data. Laboratory results, current medications, co-morbidities, relevant previous medical and medication histories were collected using data abstraction format from medical chart review. Three medical doctors and two clinical pharmacists were recruited for data collection; one clinical pharmacist was assigned to supervise the data collection process. DRP registration format was used to identify and record different types of DRPs [16]. To assure the consistency of the data collection tool, it was pretested at nearby hospital called Bedele general hospital prior to normal data collection.

### Data processing and analysis

The data were entered into a computer using *Epidemiological Information *(Epi Info) 7.2.1. The principal investigators had daily checked and clean the data. The data were then exported to statistical software for social sciences (SPSS) 24.0 for analysis. Multivariable logistic regression was used to analyze the variable by using crude odds ratio (COR) and adjusted odds ratio (AOR) with 95% CI. All variables associated with the drug-related problems at a P-value ≤ 0.25 on the bivariate analysis were entered into a multivariable logistic regression analysis to control for confounders. Finally, the predictors of drug-related problems were declared if a P value of ≤ 0.05.

### Operational definitions

**Table:** 

Drug-related problem: includes ADR, non-adherence, inappropriate indication and dose, and ineffective drug therapy [17] Poly-pharmacy: the daily consumption of 5 or more medications [17] Co-morbidity: patients diagnosed with two or more diseases [17] Prolonged hospital stay: if the patients stay ≥ 7 days [18]	

## Results

### Socio-demographic characteristics of participants

Over the study period, more than half of the participants 172(57.7%) were males. The median age of participants was 29 years and majorly distributed to age of 18–30 year class. Majority, 235(78.9%) of participants were single and 139 (46.6%) of were attended high school. With regard to their occupation, about two-thirds 100(33.6%) of them were students and one fourths 77(25.8%) of them were housewife. About two-thirds 100(33.6%) of them had a monthly income of greater than one thousand (Table 1).

**Table 1:** Socio-demographic characteristics of epileptic patients at ambulatory clinic of MKCSH

Variables	Category	Frequency	Percentage
Sex	Male	172	57.7
	Female	126	42.3
Age	18–30 years	182	61.07
	30–60 years	94	31.54
	> 60 years	22	7.38
Resident	Urban	125	41.9
	Rural	173	58.1
Marital status	Married	31	10.4
	Single	235	78.9
	Divorced	20	6.7
	Widowed	12	4
Level of education	Uneducated	22	7.4
	Elementary	98	32.9
	High school	139	46.6
	Diploma	19	6.4
	Degree	20	6.7
Occupation	Farmer	77	25.8
	Trader	41	13.8
	Gov’nt employee	30	10.1
	Student	100	33.6
	Labor worker	40	13.4
	Other *	10	3.4
Monthly income (Ethiopian Birr)	Less than 500	123	41.3
	500–1000	75	25.2
	Greater than 1000	100	33.6

Others* includes house wife and non-governmental organization employee

### Clinical characteristics of the epileptic patients

The most type of seizure was absence seizure 85(28.5%) and generalized tonic–clonic (GTC) which accounts 83 (27.9%). About half 138(46.5%) of them had a seizure frequency of greater than four whereas three-fourths 222(74.5%) of them had the epilepsy duration of less than 3 years. Nearly two-thirds 194(65.1) of the patients have no family history of epilepsy and 92(30.9%) had comorbidity (Table 2).

**Table 2:** The clinical characteristics of epileptic patients at ambulatory clinic of MKCSH

Variables	Category	Frequency	Percentage
Frequency of seizure	< 3	160	53.7
	> 4	138	46.3
Duration of epilepsy	< 3 years	222	74.5
	3—5 years	10	3.4
	> 10 years	66	22.1
Type of seizure	General tonic clonic	83	27.9
	Focal	67	22.5
	Absence seizure	85	28.5
	Unclassified	63	21.14
Family history	Yes	104	34.9
	No	194	65.1
Co-morbidity	Yes	92	30.9
	No	206	69.1

### Prevalence and types of DRPs

The prevalence of actual or potential DTPs among subjects put on at least a single drug was found to be 164(55.0%). A total of 323 DRPs were identified on average, 1.084 DRPs per patient. Poly-pharmacy was reported among 70(23.5%) of the epileptic patients. The three-leading category of drug-related problems found to be a culprit among the sample were needs additional drug therapy 72(22.29%), dose too low 52(16.09%) and dose to high which accounts 50(15.48%) (Table 3).

**Table 3:** Types of drug therapy problems among epileptic patients at  ambulatory clinic of MKCSH

Types of drug therapy problems	Frequency (*n*)	Percentage (%)
Ineffective drug therapy	42	13.00
Non-adherence	48	14.86
Dose too high	50	15.48
Needs additional drug therapy	72	22.29
Dose too low	52	16.09
Unnecessary drug therapy	49	15.17
ADR	10	3.09

### The individual drugs involved in DRPs

The most anti-epileptic drug (AED) associated with drug therapy problems were phenobarbitone 66(30.0%), carbamazepine 58(26.36%), and valproic acid 53(24.09%) (Table 4).

**Table 4:** Common AED associated with the occurrence of DRPs among epileptic patients at ambulatory clinic of MKCSH

Individual drugs	Frequency (*n*)	Percentage (%)
Phenobarbitone	66	30.0
Carbamazepine	58	26.36
Valproic acid	53	24.09
Phenytoin	43	19.55

### Determinants of medication-related problems among epileptic patients

The output of the multivariable logistic regression showed that a significant association was observed between the sex, frequency of the seizure, and poly-pharmacy with the presence of DRPs. Being a female were 3.57 times more likely to had at least one drug therapy problems than males (AOR = 3.57, 95% CI:1.85–6.88, P ≤ 0.001). Patients having frequent seizures were 2.74 more likely to have drug-related problems (AOR = 2.47, 95% CI:1.33–4.61, *P* = 0.004). Lastly, patients who have prescribed 5 or more drugs (poly-pharmacy) were 3.57 times more likely to have DRPs than patients prescribed with less than 5 drugs (AOR = 3.57, 95% CI: 1.49–8.5, *P* = 0.004) (Table 5).

**Table 5:** Bivariable and multivariable logistic regression analysis result of factors associated with DRPs among epileptic patients at ambulatory clinic of MKCSH

Variables	Category	DRPs		COR (95% CI)	AOR (95% CI)	P-value
		No (*n* = 134)	Yes (*n* = 164)			
Age	18–30	82 (61.19%)	100 (60.98%)	1	1	0.08
	31–60	44 (32.84%)	50 (30.49%)	0.932(0.57–1.54)	1.475 (0.796–2.73)	0.22
	> 60	8 (5.97)	14(8.54%)	1.44(0.57–3.59)	0.274 (.066–1.14)	0.074
Sex	Female	31 (23.13%)	95 (57.93%)	4.575(2.755–7.59)	3.57 (1.85–6.88)	< 0.001
	Male	103 (76.87%)	69 (42.07%)	1	1	
Residency	Urban	43 (32.09%)	82 (50%)	2.116(1.317–3.40)	1.09 (0.60–1.98)	0.78
	Rural	91 (67.91%)	82 (50%)	1	10.762 (0.395–1.47)	
Frequency of seizure	≤ 3	79 (58.96%)	81 (49.39%)	1	1	0.004
	> 4	55 (41.04%)	83 (50.71%)	1.47 (0.93–2.33)	2.474 (1.329–4.61)	
Family history	No	77 (57.46%)	117 (71.34%)	1	1	0.24
	Yes	57 (42.54%)	47 (28.66%)	1.843 (1.14–2.98)	1.477 (0.775–2.81)	
Poly-pharmacy	No	114 (85.07%)	114 (69.51%)	1	1	0.004
	Yes	20 (14.93%)	50 (30.49%)	2.50(1.4–4.47)	3.57 (1.496–8.5)	
Presence of comorbidity	No	89 (66.42%)	117 (71.34%)	1	1	0.36
	Yes	45 (33.58%)	47 (28.66%)	1.26(0.769–2.06)	1.21 (0.93–3.41)	

The comparisons of the pattern medication-related problems among different settings of Ethiopian hospitals revealed that the prevalence of medication-related problem was lower than the study of Tikur Anbessa Specialized Hospital (TASH) and Jimma University Medical Center (JUMC). Phenobarbitone was the commonly prescribed AEDs in all hospitals of TASH, University of Gondar Referral Hospital (UOGRH) and JUMC. The presences of medication were predictors of medication-related problem in all hospitals of TASH, UOGRH and JUMC (Table 6).

**Table 6:** The comparisons of the patterns of medication-related problem across Ethiopia hospitals

Variables	MKCSH	TASH	JUMC	UOGRH
Prevalence of DRP	55.0%	70.4%	70.0%	–
Commonly prescribed drugs	Phenobarbitone	Phenobarbitone	Phenobarbitone	Phenobarbitone
Common seizure	Absence	GTC	GTC	GTC
Common DRP	Needs additional drug therapy	ADR	Non-compliance	–
Predictors	Poly-pharmacy	Poly-pharmacy	Poly-pharmacy	–

## Discussion

The occurrence of DRPs among epileptic patients could be influenced by different factors like the number of drugs (taking ≥ 5), types of medical conditions, poly-pharmacy, female sex and history of admission to the hospital [9]. Therefore, this study tried to identify the magnitudes and determinants of this DRP among epileptic patients on chronic follow-up.

The prevalence of DRP in our study patients was 164(55.0%) which was lower than the study done in Wollega university referral hospital (71.51%) [19] and Tikur anbessa specialized hospital 70.4% [1]. The difference in magnitudes of medication-related problem observed across different settings might be due to a variation in medication therapy management classifications and study settings. Despite this difference observed, the interventions should be done to resolve DRPs to improve patient’s treatment outcomes and future researchers should use similar DRPs classification systems to generate evidence-based recommendations.

The most commonly occurred drug therapy problems was needs additional drug therapy 72(22.29%) in our study area which was different from the study of Tikur anbessa specialized hospital in which adverse drug reaction was mostly reported [1]. Similar finding was obtained from Wollega university referral hospital [19]. The variety of reports might be due to the differences in the classification of drug-related problems in different settings.

Identifying factors contributing to drug therapy problems is crucial for the reduction of unwanted effects of DRPs in epileptic patients [19]. In our study being a female were 3.57 times more likely to had at least one drug therapy problems than males. This was contrary to the study of India and Tikur Anbessa Specialized Hospital, [1, 20]. This might be due to the non-adherences of female patients towards AEDs due to fear of harm to their fetus.

The presence of comorbidity was a predictor of drug-related problem in the study of Belayneh et al. [21]. Despite this, we could not find any association between drug-related problem and the presence of comorbidity in our study area.

Patients having frequent seizures were 2.74 more likely to have drug-related problems. This was in line with the reports of University of Gondar referral hospital [22]. This finding strongly suggests that patients having frequent seizure attacks were less adherent to their medications possibly by decreasing the patients belief on medication effectiveness.

Lastly, patients who have prescribed 5 or more drugs (poly-pharmacy) were 3.57 times more likely to have DRPs than patients prescribed with less than 5. This was similar with the reports of University of Gondar referral hospital and Tikur Anbessa Specialized Hospital and Jimma university medical center [1, 22, 23]. This might be patients prescribed a poly-pharmacy could have a drug–drug interactions with other medications that result in suboptimal dosages.

## Strength and limitations

As strength, the DTPs were identified based on Cipolle DTPs classification system and Micromedex® was used as drug interaction checker and optimal sample size was obtained. As limitation, the result of the study may not be generalizable to all hospitals because it was a single-center study and the causal effect relationship was not assessed due to the retrospective nature of the study.

## Conclusion

In conclusion, absence seizure was the commonest seizure type and phenobarbitone was the most prescribed AEDs. More than half of the patients had a drug therapy problem. Needs additional drug therapy was the top ranking DTP followed by dose too low. Number of medications taken by the patients, gender and the seizure frequency had a significant association with occurrence of DTPs. Therefore, the pharmaceutical care in general and drug information services in particular should be established to tackle any undesirable medication effects in our study area. Besides this, the prescriber should check on the possibility of drug interaction with different AEDs and unnecessary combination therapy should be avoided while a mono-therapy was preferred in our study settings.

## Data Availability

The materials used while conducting this study are obtained from the corresponding author on reasonable request.

## Abbreviations

ADR: Adverse drug reaction
AED: Antiepileptic drug
AOR: Adjusted odds ratio
COR: Crude odds ratio
DRP: Drug-related problem
DTP: Drug therapy problem
JUMC: Jimma University Medical Center
MKSCH: Mettu Karl Comprehensive Specialized Hospital
TASH: Tikur Anbessa Specialized Hospital
UOGRH: University of Gondar Referral Hospital

## Declarations

### Provenance and peer review

Not commissioned, externally peer-reviewed.
